# Identifying Frailty Risk in Older Adults: The Predictive Value of Functional Tests and Center-of-Pressure-Based Postural Metrics

**DOI:** 10.3390/jcm14176266

**Published:** 2025-09-05

**Authors:** Hammad S. Alhasan

**Affiliations:** Department of Medical Rehabilitation Sciences, Faculty of Applied Medical Sciences, Umm Al-Qura University, Mecca 24382, Saudi Arabia; hshasan@uqu.edu.sa

**Keywords:** frailty, postural control, center of pressure, balance, Timed Up and Go, fear of falling, older adults, force platform, geriatrics, physiotherapy

## Abstract

**Background/Objectives**: Frailty is a multidimensional syndrome characterized by diminished physiological reserves, reduced mobility, and increased fall risk. While clinical assessments are commonly used to screen for frailty, they may not capture minor deficits in postural control. Center-of-pressure (CoP) metrics from force plates provide objective markers of postural control, yet their role in frailty screening remains underexplored. This study aimed to investigate the associations between functional performance measures and CoP-based metrics to identify predictors of frailty among older adults. **Methods**: Eighty-three adults aged ≥ 55 years with a history of falls were classified as frail or pre-frail based on modified Fried criteria. Functional assessments (Timed Up and Go (TUG), grip strength, Berg Balance Scale [BBS], Falls Efficacy Scale [FES]) and CoP metrics (mean velocity, sway path; eyes open/closed) were evaluated. Both unadjusted and age-adjusted logistic regression models were used to identify independent predictors of frailty. **Results**: Increased TUG time and number of falls were the strongest risk factors for frailty, while increased sway path and CoP velocity were protective. In particular, sway path under eyes-closed conditions showed the strongest protective association (OR = 0.323, *p* < 0.001). Additionally, fear of falling (OR = 1.078, *p* = 0.013) emerged as a significant psychological factor, consistently associated with increased frailty risk regardless of physical performance. Correlation analysis supported these findings, showing that better functional performance was linked to lower frailty risk. **Conclusions**: CoP sway path and mean velocity independently predict frailty status and offer added value beyond traditional clinical tools. These findings highlight the importance of incorporating instrumented balance assessments into frailty screening to capture nuanced postural control deficits and guide early intervention strategies.

## 1. Introduction

Frailty is a multidimensional syndrome recognized by declined reserves across multiple physiological systems, defined by phenotypic criteria such as weakness, slow gait, low physical activity, exhaustion, and unintentional weight loss [1]. The prevalence of frailty increases with age, impacting around 12–24% of community-dwelling adults, and is observed in 10–15% of individuals aged 50–64 years [2,3]. Key risk factors include advanced age, female sex, sedentary lifestyle, sarcopenia, chronic comorbidities, low grip strength, slow gait, and poor balance function [4,5]. As life expectancy increases, so does the prevalence of frailty, which is strongly associated with falls, disability and mortality [6]. Consequently, early detection of frailty is vital to hinder its progression and reduce its health and economic burden [7,8]. This study is situated within the expanding universal effort to conceptualize and manage frailty as a critical public health challenge linked with aging [9,10,11,12].

Functional clinical outcomes including the Timed Up and Go (TUG), Berg Balance Scale (BBS), and Falls Efficacy Scale (FES) play a key role in frailty assessment by providing insight into physical, functional and psychological aspects of mobility and balance. The TUG test has demonstrated robust predictive validity around 0.72 (95% CI 0.67–0.76; *p* < 0.001) for frailty among older populations and effectively distinguishing frail from non-frail individuals [10]. Likewise, the BBS strongly correlates with fall risk and frailty; lower BBS scores consistently align with reduced stability and higher frailty risk [11]. Subjective scale such as the FES reflect fear of falling and confidence in maintaining balance during tasks [12]. Higher scores on the FES have been linked to declined physical performance and higher fall incidence [13]. This relationship highlights a feedback loop where fear of falling leads to activity limitation, deconditioning, and accelerated frailty progression. This study is consistent with the phenotypic model of frailty outlined by Fried et al., which addresses observable clinical and physical deficits [14]. However, given the multidimensional nature of frailty, adding psychological and postural factors such as fear of falling and balance instability adds conceptual depth to the assessment.

Center-of-pressure (CoP) metrics are quantitative measures of body sway derived from force plates during standing tasks [15]. They represent the adjustments made by the neuromuscular system to maintain standing position [16]. Common CoP parameters include sway path length and mean velocity, that illustrate the magnitude and speed of postural alterations, respectively [17]. While functional clinical outcomes are well-documented, they may overlook more subtle deteriorations in postural control or physiological resilience [18,19]. Thus, recent studies have started to verify a link between center-of-pressure (CoP) metrics and frailty status, implying that postural control measured via force platforms can potentially provide objective markers of physiological resilience. For instance, a study reported that non-frail older adults showed significantly better postural control as identified by lower CoP velocity and reduced sway when compared to frail older adults [20]. Furthermore, a study comparing frail and pre-frail older adults reported that reduced CoP velocity was significantly associated with greater frailty, suggesting that lower CoP movement may reflect poor postural control [21]. Additionally, a systematic review reported that CoP metrics such as sway area and mean velocity are strong predictors of fall risk in older adults, confirming their potential relevance to frailty [22]. Similarly, a wearable insole system and CoP metrics can detect frailty in older adults with 75% accuracy; it shows potential as an accessible method for early frailty assessment based on gait and balance measures [23].

These findings strengthen the concept that moderate CoP sway and velocity are indicative of optimum postural control mechanisms, while minimum CoP sway and velocity may reflect the stiffness and neuromuscular decline intrinsic in frailty [24]. Recent research continues to support the significance of CoP metrics as objective indicators of postural control in older adults. For instance, Jafari et al. (2023) [25], explored the use of CoP to classify balance impairments in older adults during static standing. They identified three distinct postural control profiles. Notably, participants with greater dependance on high-frequency CoP components exhibited weaker balance and increased fear of falling. These findings highlight the potential of CoP metrics particularly in the frequency domain. However, Jafari et al.’s work did not integrate commonly used clinical frailty measures and their analysis focused exclusively on frequency-based CoP metrics, without exploring the use of amplitude-based metrics such as mean CoP velocity and sway path length. Nevertheless, few studies have compared amplitude-based CoP metrics across frailty stages using a integrated clinical and posturographic approach. Additionally, the contribution of psychological indicators such fear of falling has rarely been considered. The current study addresses these gaps by assessing whether traditional amplitude-domain CoP parameters can serve as independent predictors of frailty when considered together with clinical, functional, and psychological factors. By integrating functional tests with objective CoP metrics, the current study provides a more comprehensive approach to frailty assessment. This study makes an original contribution by integrating functional tests, psychological factors, and CoP metrics into a single analytical model for frailty risk. Contrary to previous studies that examined clinical or postural measures separately, our approach provides a more comprehensive framework for frailty assessment. Thus, this work highlights the novel role of CoP metrics as objective indicators that supplement traditional clinical tools.

In recent years, machine learning techniques have been growingly adopted to predict chronic disease risk by incorporating broad clinical, physiological, and lifestyle datasets. For example, machine learning algorithms can detect complex, nonlinear relationships between multiple predictors, providing high predictive accuracy in identifying at-risk populations [26]. While these methods are promising, they necessitate larger datasets, and external validation before clinical translation [27]. In contrast, this study employs traditional logistic regression models, which provide interpretable effect estimates (odds ratios) that are clinically intuitive and directly applicable in routine practice.

Therefore, the purpose of this study was to explore the association between functional performance measures and CoP metrics with frailty status in older adults. This study aimed to identify key clinical and postural control predictors of frailty in older adults by exploring the relationship between functional outcomes (e.g., TUG, grip strength, BBS, FES) and instrumented balance metrics (mean CoP velocity and sway path) using force plate assessments. The objectives are as follows: (1) to compare clinical performance measures (TUG, grip strength, BBS) and instrumented balance metrics (Mean Velocity Eyes Open/Eyes Closed, Sway Path Eyes Open/Eyes Closed) between pre-frail and frail older adults; (2) to determine the strength and direction of associations between clinical and posturography-based balance metrics using correlation analysis; and (3) to identify which variables independently predict frailty status after controlling for age through logistic regression modeling. The significance of this work is situated in its potential to promote early detection of frailty by capturing nuanced declines in postural control and mobility that traditional tests may miss. This approach not only adds crucial data but also evaluates the practical application of CoP metrics in frailty risk stratification, potentially guiding future assessment and intervention strategies.

## 2. Materials and Methods

### 2.1. Ethical Approval and Consent to Participate

This was a cross-sectional observational study. The study protocol was approved by the Ethics Committee of Umm Al-Qura University (Approval No. HAPO-02-K-012-2025-11-2337). All participants provided written informed consent prior to enrollment, in accordance with the Declaration of Helsinki. Participant anonymity and data confidentiality were ensured throughout the research process.

### 2.2. Participants and Eligibility Criteria

**Figure 1:**
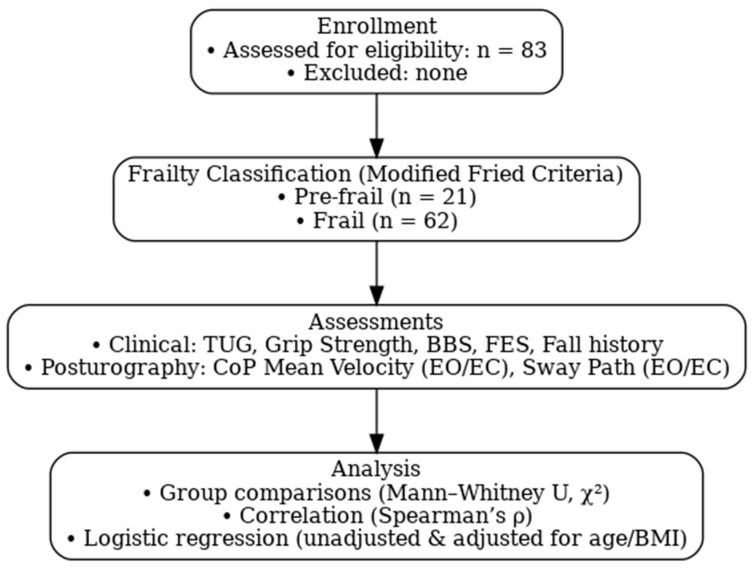
Flowchart of the current study.

A total of 83 community-dwelling adults aged 55 years and older were recruited using a convenience sampling approach from local health centers, senior centers, participant referrals, flyers or posters and social media platforms in Saudi Arabia. Inclusion criteria were (1) age ≥ 55 years, (2) Self-reported history of one or more falls within the past 12 months, and (3) ability to stand unassisted for at least 30 s. Exclusion criteria included (1) Diagnosed neurological disorders known to impair balance or gait, (2) severe musculoskeletal impairment, (3) uncorrected vision problems, and (4) cognitive impairment that limited the ability to follow instructions. Frailty categorization was based on clinical assessment and included individuals categorized as frail or pre-frail. The frailty categorization method used was derived from the Fried phenotype criteria. These criteria were adopted to ensure the inclusion of individuals at risk of frailty while minimizing confounding factors that could independently impair balance, mobility, or cognition. The study flowchart (Figure 1) summarizes participant enrollment, frailty classification, assessments, and analysis steps.

### 2.3. Outcome Measures

Participants completed the following standardized clinical tests.

#### 2.3.1. Frailty Index

This was assessed using a modified version of the Fried Phenotype Criteria, which includes five components: self-reported unintentional weight loss (≥4.5 kg in the past year), self-reported exhaustion, self-reported low physical activity level, slowness (assessed via TUG), and weakness (assessed via grip strength). Participants meeting three or more criteria were classified as frail, while those meeting one or two criteria were classified as pre-frail.

#### 2.3.2. Physical Function

i. Grip Strength: Measured by a calibrated Jamar hydraulic hand dynamometer. Participants completed three maximal trials with the dominant hand, and the highest value (kg) was recorded.
ii. Timed Up and Go (TUG) [28]: Participants were directed to stand up from a chair, walk 3 m, turn around, return, and sit down. The time to complete the task was recorded.
iii. Berg Balance Scale (BBS) [29]: A 14-item scale assessing functional balance, scored from 0 to 56, with lower scores indicating impaired balance.
iv. Falls Efficacy Scale (FES-I) [30]: A 10-item questionnaire evaluating fear of falling during activity of daily living. Higher scores indicate greater fear and lower balance confidence.
v. Fall History: Participants self-reported the number of falls experienced in the previous 12 months.

### 2.4. Posturography and Force Plate Measures

This study followed general recommendations for stabilometry research [31]. Static postural control was assessed using an AMTI force platform. Participants stood barefoot in a bipedal stance for 60 s under two conditions: Eyes Open (EO) and Eyes Closed (EC) Data were sampled at 100 Hz and filtered using a 4th-order low-pass Butterworth filter at 10 Hz. The following center-of-pressure (CoP) metrics were extracted for each trial: Mean CoP Velocity in cm/s and Sway Path Area in cm^2^. All CoP values represent averages across trials. Data processing and calculation were performed using custom MATLAB (Version R2019a) scripts.

### 2.5. Sample Size Calculation

A priori sample size calculation was performed using G*Power software (version 3.1.9.7) to estimate the minimum number of individuals needed for binary logistic regression. Assuming a medium effect size (odds ratio = 2.0), α = 0.05, statistical power (1−β) = 0.80, and a two-tailed test, the required total sample size was estimated to be 84 participants. This estimate also fulfills the recommended rule of at least 10 events per predictor variable in multivariate logistic models. The final sample of 83 participants is in close agreement with this estimate and was deemed sufficient to detect significant associations between clinical/posturographic predictors and frailty status.

### 2.6. Statistical Analysis

All analyses were performed using IBM SPSS Statistics version 27.0 (IBM Corp., Armonk, NY, USA). Descriptive statistics (means, standard deviations, percentages) were calculated for all variables. Normality of continuous data was assessed using the Shapiro–Wilk test. Non-parametric methods were employed throughout the analysis. Mann–Whitney U tests were used for group comparisons (frail vs. pre-frail) and chi-square tests were used for categorical variables. Spearman’s rank-order correlation was used to explore relationships between frailty-related clinical measures and CoP outcomes. Correlation coefficients (ρ) were interpreted as follows: values between ±0.10 and ±0.29 were considered weak, ±0.30 to ±0.49 moderate, ±0.50 to ±0.69 strong, and ±0.70 to ±1.00 very strong, with statistical significance set at *p* < 0.05.

Binary logistic regression was used to detect predictors of frailty status. Unadjusted (bivariate) models were first computed for each variable followed by adjusted models controlling for age. Odds ratios (OR), 95% confidence intervals (CI), and *p*-values were reported. Variables that remained significant in adjusted models were interpreted as independent predictors.

## 3. Results

The sample included 83 participants with a mean age of 65.35 ± 10.72 years. Mean height and weight were 165.58 ± 8.87 cm and 77.36 ± 13.24 kg, respectively, resulting in a mean BMI of 28.02 ± 5.51 kg/m^2^. Participants had an average grip strength of 28.57 ± 7.17 kg and reported an average of 2.08 ± 1.78 falls. Functional assessments showed a mean TUG time of 13.43 ± 2.27 s, a BBS score of 44.54 ± 5.98, and an FES score of 25.01 ± 12.69, suggesting a moderate to high risk of falls on average.

74.7% (*n* = 62) of participants were classified as frail based on the Fried Phenotype Criteria. The frail group showed significantly lower outcomes across all measures compared to the pre-frail group (*p* < 0.001). Gender did not significantly differ between groups (*p* = 0.661). Similarly, BMI and weight differences were not statistically significant (*p* = 0.814 and *p* = 0.192, respectively). Table 1 presents a comparison of demographic, clinical, and posturography variables between pre-frail and frail older adults.

The normality of the variables were assessed using the Shapiro–Wilk test. The results indicated that most variables violated the assumption of normality in both groups (*p* < 0.05). Given these results Mann–Whitney U test were employed for group comparisons between frail and pre-frail participants.

### 3.1. Correlation Between Clinical Measures and Postural Control

Spearman’s correlation analysis was performed to examine the relationships between clinical assessments and objective posturography. The results demonstrated several statistically significant correlations (see Table 2).

Higher TUG scores were moderately associated with reduced CoP velocity (ρ = −0.645 for eyes open; −0.620 for eyes closed) and sway path (ρ = −0.393 and −0.336, respectively), suggesting more rigid postural control.

BBS scores showed strong positive correlations with both CoP velocity (ρ = 0.803, 0.720) and sway path (ρ = 0.681, 0.565), indicating that better balance performance is linked to more dynamic postural behavior.

Higher FES scores were moderately associated with reduced CoP velocity (ρ = −0.395, −0.371) and sway path (ρ = −0.348, −0.343), reflecting a cautious, less adaptive strategy. Similarly, greater fall frequency was moderately to strongly correlated with lower CoP velocity (ρ = −0.597, −0.537) and sway path (ρ = −0.422, −0.427), indicating constrained balance control in frequent fallers.

### 3.2. Unadjusted Logistic Regression

Table 3 summarizes the unadjusted and adjusted logistic regression results, identifying key predictors of frailty.

The number of falls emerged as the strongest risk factor for frailty, with each additional fall increasing the odds by 265.2% (OR = 3.652, *p* < 0.001). This was followed by TUG, where each 1 s increase was associated with a 160.7% higher frailty risk (OR = 2.607, *p* < 0.001).

Grip strength also showed a protective effect, with each 1 kg increase reducing frailty odds by 19.9% (OR = 0.801, *p* < 0.001). Additional protective factors included FES which was a weaker but significant predictor, with each unit increase raising frailty odds by 7.8% (OR = 1.078, *p* = 0.013).

Among the protective factors, SP_EC (sway path with eyes closed) showed the greatest reduction in frailty odds with each unit increase in sway path decreasing the odds of frailty by 67.7% (OR = 0.323, *p* < 0.001), followed by SP_EO (OR = 0.432), MV_EC (OR = 0.597), and MV_EO (OR = 0.635).

### 3.3. Adjusted Logistic Regression

In the adjusted logistic regression analysis controlling for age, SP_EC (sway path with eyes closed) remained the strongest protective factor against frailty. Each unit increase in SP_EC was associated with a 67.7% reduction in the odds of being frail (OR = 0.323, *p* < 0.001). This was followed by SP_EO (OR = 0.432, *p* < 0.001), MV_EC (OR = 0.597, *p* < 0.001), and MV_EO (OR = 0.635, *p* < 0.001)—all indicating that greater sway or velocity measures from posturography were significantly associated with lower frailty risk.

Functional performance measures also showed strong associations. TUG was the most prominent risk factor, with each additional second increasing frailty odds by 160.7% (OR = 2.607, *p* < 0.001). Similarly, Number of Falls remained a strong predictor, with each additional fall raising frailty odds by 265.2% (OR = 3.652, *p* < 0.001).

Grip Strength continued to demonstrate a substantial protective effect, where each 1 kg increase reduced frailty risk by 19.9% (OR = 0.801, *p* < 0.001). Additional protective functional indicators included BBS (OR = 0.806, *p* < 0.001), while FES was the weakest but still significant risk factor, with each point increase associated with a 7.8% rise in frailty odds (OR = 1.078, *p* = 0.013).

Detailed model diagnostics, including pseudo R^2^ values, goodness-of-fit tests, and classification accuracy for all logistic regression models are provided in Supplementary Materials.

## 4. Discussion

This study showed that both clinical performance measures and CoP metrics are significantly associated with frailty status in older adults. Particularly sway path and CoP velocity under eyes-open and eyes-closed conditions were identified as strong protective factors against frailty, even after adjusting for age. Functional measures such as TUG and number of falls were the strongest risk factors, while grip strength BBS and FES were significant protective indicators. These findings highlight the importance of integrating both clinical and objective balance assessments when evaluating frailty.

Regarding CoP metrics, this study found that higher mean CoP velocity and greater sway path length were significantly associated with lower odds of frailty after adjusting for age. These findings oppose the common interpretation that increased sway indicates poor balance and instead facilitate a more detailed understanding of postural control in older adults. While increased CoP velocity is commonly used as an index of impaired postural control, some studies advocate that lower velocity may indicate reduced postural responsiveness rather than enhanced balance [32,33]. Compared to pre-frail individuals, frail individuals exhibit reduced CoP movement due to stiffness, guarding behavior, or fear of falling, which may limit postural adjustments [21]. A similar pattern was noted for sway path length, while greater sway is commonly reported as a sign of instability [24,34], several studies have shown that frail individuals may adopt a conservative strategy by lowering sway through co-contraction of the ankle joints [25,35]. Moreover, a lifespan analysis of postural dynamics found that older adults showed more constrained CoP behavior, suggesting decreased responsiveness and adaptive capacity [36]. These insights together support a more refined interpretation of postural control in frail individuals, suggesting that both excessive and reduced CoP movement can be maladaptive, and that effective postural control may be reflected in a dynamic, responsive sway pattern rather than minimal displacement alone.

In both regression models, TUG and number of falls were consistently associated with elevated risk of frailty. These findings are in line with prior literature indicating that slow gait speed and fall history are significant indicators of declining physical function [10]. Importantly, after adjusting for age the effect of number of falls on frailty was amplified, with individuals who reported more falls showing over a sevenfold increase in frailty risk. Supporting this, findings from meta-analyses highlight fall history as a strong independent predictor of both future frailty and risk of falls [37]. This emphasizes the significance of fall prevention strategies in frailty management. Regarding grip strength, it was significantly protective in the unadjusted model. However, its impact minimized after adjustment for age (*p* = 0.055), suggesting that age may in part influence the association between muscle strength and frailty. This attenuation is consistent with a longitudinal study analysis that demonstrates that low grip strength is a core component of frailty, yet its predictive power may be reduced when accounting for age and body composition [38]. Furthermore, a recent longitudinal study using machine learning models identified grip strength as an independent predictor of frailty, although other factors such as age, BMI, and cognitive ability were identified as a stronger predictor [39].

In contrast, fear of falling was significantly associated with increased risk of frailty in both models, this finding highlights the growing recognition that frailty is not only a physical condition but one deeply altered by psychological and behavioral factors [5]. Fear of falling, while considered a psychological reaction to poor postural control, has consistently been shown to initiate a series of functional declines resulting in activity restriction, muscle atrophy and reduced mobility, each of which promotes frailty progression [40,41]. A recent longitudinal study reported that older adults who limit their activity due to fear of falling had significantly higher frailty incidence, regardless of baseline health status [42]. This suggests that fear of falling is more than a secondary sign of frailty; it is a principal process in the development of frailty. Its consistent predictive value across both models underscores the need to regularly assess fear of falling in frailty screening protocols. From a clinical perspective, addressing FoF may not only alleviate risk but also improve outcomes in frailty interventions. In summary, these results highlight the value of combining clinical measures (e.g., TUG, grip strength) with objective postural control assessments to distinguish individuals at risk of frailty. Moreover, the findings suggest that not all traditional markers of postural stability (e.g., reduced sway) are beneficial, context and physiological adaptability must also be considered.

This study has several limitations that should be mentioned. First, the cross-sectional design limits the ability to determine causality between frailty status and the associated clinical and CoP measures. Though significant associations were observed, longitudinal studies are needed to determine temporal associations and the predictive value of CoP metrics over time. Second, the sample size, specifically the pre-frail subgroup, was relatively small, which may have minimized statistical power and elevated the risk of type II errors in subgroup analyses. Future studies with larger sample size are warranted to validate these findings and improve generalizability. Third, the frailty classification was informed by the Fried phenotype criteria, which may not reflect the full complex construct of frailty, especially psychological or social components. Adopting more comprehensive frailty evaluation tools can offer a more inclusive understanding of the condition. Fourth, while this study incorporated validated functional outcome measures, other related variables such as nutritional status, cognitive function, or comorbidity burden were not evaluated. These may have altered both frailty and postural control outcomes and should be added in future models to better control for potential confounders. Fifth, the force plate assessments focused on static conditions only; dynamic balance tasks may offer further insights into adaptive postural responses that static conditions lack the capacity to fully capture. Lastly, while logistic regression was adopted in this study for its clinical applicability, future research with a larger sample size should compare its performance against more advanced machine learning models to establish whether predictive accuracy can be enhanced while maintaining clinical utility.

From a clinical perspective, these findings underscore the value of integrating both functional performance tests and instrumented postural assessments into frailty screening protocols. Traditional measures such as TUG and grip strength remain strong predictors of frailty, while CoP-based metrics offer objective insights into subtle postural control impairment that may precede explicit functional decline. Incorporating CoP metrics coupled with clinical tools could facilitate earlier identification of individuals at risk and support more individualized rehabilitation strategies. Furthermore, the consistent role of fear of falling underscores the significance of addressing psychological and physical domains in frailty management.

## 5. Conclusions

This study provides evidence that both clinical and posturographic measures are independently associated with frailty in older adults. TUG and fall history emerged as strong predictors, while grip strength, BBS and FES were protective. Importantly, CoP sway path length and mean velocity demonstrated significant inverse associations with frailty. These findings support the integration of objective postural control assessments alongside conventional clinical tools to enhance early identification and risk stratification of frailty. Our findings contribute new evidence that more dynamic CoP behavior may reflect adaptability rather than impairment, challenging conventional interpretations of postural control in frailty.

The innovative aspect of this study lies in demonstrating that greater CoP sway and velocity, often considered signs of instability, may instead reflect adaptability and resilience in postural control among older adults. By combining conventional clinical measures with objective CoP-based assessments and psychological factors, this study provides new evidence for a multidimensional and clinically applicable approach to frailty risk stratification.

## Figures and Tables

**Table 1:** Baseline characteristics of included participants.

Variable	Pre-Frail (*n* = 21)	Frail (*n* = 62)	*p*-Value
Age (years)	54.29 (9.44)	69.10 (8.30)	<0.001
Gender (% male)	33.3%	42.0%	0.661
Height (cm)	158.95 (8.70)	167.82 (7.80)	<0.001
Weight (kg)	76.48 (16.40)	77.65 (12.21)	0.192
BMI (kg/m^2^)	29.02 (7.05)	27.68 (4.90)	0.814
Grip Strength (kg)	35.73 (6.73)	26.14 (5.52)	<0.001
No. of Falls	0.24 (0.44)	2.71 (1.61)	<0.001
TUG (s)	11.74 (1.04)	14.00 (2.29)	<0.001
BBS	49.84 (6.47)	42.74 (4.63)	<0.001
FES	19.13 (18.05)	27.00 (9.69)	<0.001
MV_EO (cm/s)	2.81 (2.75)	9.16 (3.77)	<0.001
MV_EC (cm/s)	2.97 (2.58)	8.26 (2.85)	<0.001
SP_EO (cm^2^)	3.14 (1.36)	5.81 (2.16)	<0.001
SP_EC (cm^2^)	3.19 (1.24)	5.26 (1.45)	<0.001

**Table 2:** Spearman’s correlation coefficients (ρ) between clinical measures and posturography-based CoP metrics.

Clinical Measure	Posturography Variable	ρ (Spearman)	95% CI (Lower–Upper)	*p*-Value
TUG	MV_EO	−0.645	[−0.758, −0.494]	<0.001
	MV_EC	−0.620	[−0.748, −0.456]	<0.001
	SP_EO	−0.393	[−0.610, −0.146]	0.001
	SP_EC	−0.336	[−0.566, −0.036]	0.002
BBS	MV_EO	0.803	[0.693, 0.882]	<0.001
	MV_EC	0.720	[0.593, 0.812]	<0.001
	SP_EO	0.681	[0.523, 0.795]	<0.001
	SP_EC	0.565	[0.366, 0.715]	<0.001
FES	MV_EO	−0.395	[−0.611, −0.149]	<0.001
	MV_EC	−0.371	[−0.594, −0.122]	0.001
	SP_EO	−0.348	[−0.579, −0.093]	0.001
	SP_EC	−0.343	[−0.570, −0.088]	0.001
No of Falls	MV_EO	−0.597	[−0.732, −0.432]	<0.001
	MV_EC	−0.537	[−0.678, −0.352]	<0.001
	SP_EO	−0.422	[−0.629, −0.221]	<0.001
	SP_EC	−0.427	[−0.632, −0.226]	<0.001

**Table 3:** Comparison of unadjusted and adjusted logistic regression models predicting frailty status, adjusted for age.

Predictor Variable	Unadjusted OR (95% CI)	*p*-Value	Adjusted OR (95% CI)	*p*-Value
MV_EO	0.747 (0.593–0.941)	0.013	0.635 (0.525–0.767)	<0.001
MV_EC	0.724 (0.546–0.959)	0.024	0.597 (0.481–0.740)	<0.001
SP_EO	0.616 (0.408–0.931)	0.021	0.432 (0.293–0.636)	<0.001
SP_EC	0.528 (0.296–0.940)	0.030	0.323 (0.189–0.554)	<0.001
TUG	2.418 (1.302–4.488)	0.005	2.607 (1.621–4.194)	<0.001
Grip Strength	0.898 (0.804–1.002)	0.055	0.801 (0.725–0.886)	<0.001
BBS	0.913 (0.814–1.023)	0.117	0.806 (0.729–0.892)	<0.001
Number of Falls	7.234 (1.969–26.571)	0.003	3.652 (2.112–6.313)	<0.001
FES	1.053 (1.005–1.103)	0.028	1.078 (1.016–1.143)	0.013

## Data Availability

The original contributions presented in the study are included in the article; further inquiries can be directed at the corresponding author.

## Supplementary Materials

The following supporting information can be downloaded at https://www.mdpi.com/article/10.3390/jcm14176266/s1, Table S1. Adjusted Logistic Regression Model Diagnostics. Table S2. Unadjusted Logistic Regression Model Diagnostics.

## Abbreviations

The following abbreviations are used in this manuscript:

BBS	Berg Balance Scale
BMI	Body Mass Index
CI	Confidence Interval
cm/s	Centimeters per Second
CoP	Center of Pressure
EC	Eyes Closed
EO	Eyes Open
FES-I	Falls Efficacy Scale–International
kg	Kilogram
MV_EC	Mean Velocity with Eyes Closed
MV_EO	Mean Velocity with Eyes Open
OR	Odds Ratio
SP_EC	Sway Path with Eyes Closed
SP_EO	Sway Path with Eyes Open
TUG	Timed Up and Go

## References

[1] Fierro-Marrero J., Reina-Varona Á., Paris-Alemany A., La Touche R. (2025). Frailty in geriatrics: A critical review with content analysis of instruments, overlapping constructs, and challenges in diagnosis and prognostic precision. J. Clin. Med..

[2] Long Q., Li Y., Shi Z., Lee Y., Mao L. (2025). Investigation of the association between the triglyceride-glucose index and the incidence of frailty among middle-aged and older adults: Evidence from the China health and retirement longitudinal study. Front. Public Health.

[3] O’cAoimh R., Sezgin D., O’dOnovan M.R., Molloy D.W., Clegg A., Rockwood K., Liew A. (2021). Prevalence of frailty in 62 countries across the world: A systematic review and meta-analysis of population-level studies. Age Ageing.

[4] Battista F., Duregon F., Vecchiato M., Ermolao A., Neunhaeuserer D. (2025). Sedentary lifestyle and physical inactivity: A mutual interplay with early and overt frailty. Nutr. Metab. Cardiovasc. Dis..

[5] de Souza L.F., Canever J.B., Moreira B.D.S., Danielewicz A.L., de Avelar N.C.P. (2022). Association between fear of falling and frailty in community-dwelling older adults: A systematic review. Clin. Interv. Aging.

[6] Kim D.H., Rockwood K. (2024). Frailty in older adults. N. Engl. J. Med..

[7] De Luca V., Donnoli C., Formosa V., Carnevale E., Bisogno M., Patumi L., Leonardini L., Obbia P., Palummeri E., Ruatta M. (2025). Preliminary results of a multidimensional approach to screen for frailty in community-dwelling older adults of eight Italian regions: The SUNFRAIL+ study. Front. Public Health.

[8] Wang X.-M., Zhang Y.-H., Meng C.-C., Fan L., Wei L., Li Y.-Y., Liu X.-Z., Lv S.-C. (2024). Scale-based screening and assessment of age-related frailty. Front. Public Health.

[9] Dent E., Martin F.C., Bergman H., Woo J., Romero-Ortuno R., Walston J.D. (2019). Management of frailty: Opportunities, challenges, and future directions. Lancet.

[10] Wada Y., Shojima K., Tamaki K., Mori T., Kusunoki H., Onishi M., Tsuji S., Matsuzawa R., Nagai K., Sano K. (2023). Association between timed Up-and-Go test and future changes in the frailty status in a longitudinal study of Japanese Community-Dwelling older adults. Clin. Interv. Aging.

[11] Beck Jepsen D., Robinson K., Ogliari G., Montero-Odasso M., Kamkar N., Ryg J., Freiberger E., Masud T. (2022). Predicting falls in older adults: An umbrella review of instruments assessing gait, balance, and functional mobility. BMC Geriatr..

[12] McGarrigle L., Yang Y., Lasrado R., Gittins M., Todd C. (2023). A systematic review and meta-analysis of the measurement properties of concerns-about-falling instruments in older people and people at increased risk of falls. Age Ageing.

[13] McColl L., Strassheim V., Linsley M., Green D., Dunkel C., Trundle H., Gibbon J.R., Parry S.W. (2024). Associations between the Falls Efficacy Scale International (FES-I) and poor strength and balance in community-dwelling older people. Cogent Gerontol..

[14] Fried L.P., Tangen C.M., Walston J., Newman A.B., Hirsch C., Gottdiener J., Seeman T., Tracy R., Kop W.J., Burke G. (2001). Frailty in older adults: Evidence for a phenotype. J. Gerontol. A Biol. Sci. Med. Sci..

[15] Borrelli J., Komisar V., Novak A., Maki B., King E. (2020). Extending the center of pressure to incorporate handhold forces: Derivation and sample application. J. Biomech..

[16] Mongold S.J., Georgiev C., Legrand T., Yildiran Carlak E., Iannotta A., Cabaraux P., Naeije G., Vander Ghinst M., Bourguignon M. (2025). Aging-related changes in neuromuscular control strategies and their influence on postural stability. Sci. Rep..

[17] Rizzato A., Benazzato M., Cognolato M., Grigoletto D., Paoli A., Marcolin G. (2023). Different neuromuscular control mechanisms regulate static and dynamic balance: A center-of-pressure analysis in young adults. Hum. Mov. Sci..

[18] Serra-Prat M., Palomera E. (2019). Muscle strength, sarcopenia and frailty associations with balance and gait parameters: A cross-sectional study. Eur. J. Geriatr. Gerontol..

[19] Rezaei A., Bhat S.G., Cheng C.-H., Pignolo R.J., Lu L., Kaufman K.R., Jan Y.-K. (2024). Age-related changes in gait, balance, and strength parameters: A cross-sectional study. PLoS ONE.

[20] Dallaire M., Houde-Thibeault A., Bouchard-Tremblay J., Wotto E.A., Côté S., Oliveira C.S., Ngomo S., da Silva R.A. (2024). Impact of frailty and sex-related differences on postural control and gait in older adults with Parkinson’s Disease. Exp. Gerontol..

[21] Pinloche L., Zhang Q., Berthouze S.E., Monteil K., Hautier C. (2022). Physical ability, cervical function, and walking plantar pressure in frail and pre-frail older adults: An attentional focus approach. Front. Aging.

[22] Quijoux F., Vienne-Jumeau A., Bertin-Hugault F., Zawieja P., Lefèvre M., Vidal P.-P., Ricard D. (2020). Center of pressure displacement characteristics differentiate fall risk in older people: A systematic review with meta-analysis. Ageing Res. Rev..

[23] Anzai E., Ren D., Cazenille L., Aubert-Kato N., Tripette J., Ohta Y. (2022). Random forest algorithms to classify frailty and falling history in seniors using plantar pressure measurement insoles: A large-scale feasibility study. BMC Geriatr..

[24] Zhuang Y., Hong Z., Wu L., Zou C., Zheng Y., Chen L., Yin L., Qin J. (2023). Influence of age on static postural control in adults with type 2 diabetes mellitus: A cross-sectional study. Front. Endocrinol..

[25] Jafari H., Gustafsson T., Nyberg L., Röijezon U. (2023). Predicting balance impairments in older adults: A wavelet-based center of pressure classification approach. Biomed. Eng. Online.

[26] Du J., Tao X., Zhu L., Qi W., Min X., Deng H., Wei S., Zhang X., Chang X. (2025). A risk prediction system for depression in middle-aged and older adults grounded in machine learning and visualization technology: A cohort study. Front. Public Health.

[27] Oliosi E., Guede-Fernández F., Londral A. (2022). Machine learning approaches for the frailty screening: A narrative review. Int. J. Env. Res. Public Health.

[28] Podsiadlo D., Richardson S. (1991). The timed “Up & Go”: A test of basic functional mobility for frail elderly persons. J. Am. Geriatr. Soc..

[29] Berg K., Wood-Dauphine S., Williams J.I., Gayton D. (1989). Measuring balance in the elderly: Preliminary development of an instrument. Physiother. Can..

[30] Yardley L., Beyer N., Hauer K., Kempen G., Piot-Ziegler C., Todd C. (2005). Development and initial validation of the Falls Efficacy Scale-International (FES-I). Age Ageing.

[31] Scoppa F., Capra R., Gallamini M., Shiffer R. (2013). Clinical stabilometry standardization: Basic definitions–acquisition interval–sampling frequency. Gait Posture.

[32] Sarvari M., Shanbehzadeh S., Shavehei Y., ShahAli S. (2024). Postural control among older adults with fear of falling and chronic low back pain. BMC Geriatr..

[33] Promsri A., Pitiwattanakulchai P., Saodan S., Thiwan S. (2024). Age-Related Changes in Postural Stability in Response to Varying Surface Instability in Young and Middle-Aged Adults. Sensors.

[34] Schülein S., Sieber C.C., Gaßmann K.-G., Ritt M. (2020). Frail Older Individuals Maintaining a Steady Standing Position: Associations Between Sway Measurements with Frailty Status Across Four Different Frailty Instruments. Clin. Interv. Aging.

[35] Falk J., Strandkvist V., Pauelsen M., Vikman I., Nyberg L., Röijezon U. (2022). Increased co-contraction reaction during a surface perturbation is associated with unsuccessful postural control among older adults. BMC Geriatr..

[36] Van Humbeeck N., Kliegl R., Krampe R.T. (2023). Lifespan changes in postural control. Sci. Rep..

[37] Wapp C., Hager A.-G.M., Rikkonen T., Hilfiker R., Biver E., Ferrari S., Kröger H., Zwahlen M., Zysset P. (2024). Validation of a fall rate prediction model for community-dwelling older adults: A combined analysis of three cohorts with 1850 participants. BMC Geriatr..

[38] Huang L., Shen X., Zou Y., Wang Y. (2024). Effects of BMI and grip strength on older adults’ falls—A longitudinal study based on CHARLS. Front. Public Health.

[39] Yu L., Cao S., Song B., Hu Y. (2024). Predicting grip strength-related frailty in middle-aged and older Chinese adults using interpretable machine learning models: A prospective cohort study. Front. Public Health.

[40] Wu K.-Y., Chen D.-R., Chan C.-C., Yeh Y.-P., Chen H.-H. (2023). Fear of falling as a mediator in the association between social frailty and health-related quality of life in community-dwelling older adults. BMC Geriatr..

[41] Baek W., Min A., Ji Y., Park C.G., Kang M. (2024). Impact of activity limitations due to fear of falling on changes in frailty in Korean older adults: A longitudinal study. Sci. Rep..

[42] Mo C., Peng W., Luo Y., Tang S., Liu M. (2023). Bidirectional relationship between fear of falling and frailty among community-dwelling older adults: A longitudinal study. Geriatr. Nurs..

