# Supplementary material for: Identifying Frailty Risk in Older Adults: The Predictive Value of Functional Tests and Center-of-Pressure-Based Postural Metrics
Source: J Clin Med. 2025 Sep 5;14(17):6266. doi: 10.3390/jcm14176266 (PMC12429373; doi:10.3390/jcm14176266)
Supplement: Supplementary file 1 [file jcm-14-06266-s001.zip › jcm-3826763-supplementary.pdf]

This section summarizes the model strength (pseudo R^2), calibration (Hosmer–Lemeshow test), and classification performance of all adjusted and unadjusted logistic regression models used to predict frailty status.

Table S1. Adjusted Logistic Regression Model Diagnostics

Predictor Variable	Nagelkerke R^2	Hosmer–Lemeshow χ^2 (p)	Classification Accuracy (%)
**MV_EO**	0.599	9.761 (p = .282)	91.6%
**MV_EC**	0.578	8.899 (p = .351)	88.0%
**SP_EO**	0.548	7.408 (p = .492)	86.7%
**SP_EC**	0.527	10.085 (p = .258)	84.3%
**Grip Strength**	0.436	7.134 (p = .521)	84.3%
**TUG**	0.250	5.232 (p = .732)	80.7%
**FES**	0.108	4.245 (p = .835)	73.5%
**BBS**	0.083	11.362 (p = .182)	72.3%

Table S2. Unadjusted Logistic Regression Model Diagnostics

Predictor Variable	Nagelkerke R^2	Hosmer–Lemeshow χ^2 (p)	Classification Accuracy (%)
**MV_EO**	0.547	8.970 (p = .345)	85.5%
**MV_EC**	0.531	8.727 (p = .366)	80.7%
**SP_EO**	0.449	7.632 (p = .470)	85.5%
**SP_EC**	0.450	5.736 (p = .677)	80.7%
**Grip Strength**	0.436	21.814 (**p = .005**)	80.7%
**TUG**	0.396	11.340 (p = .183)	74.7%
**BBS**	0.349	15.228 (p = .055)	78.3%
**FES**	0.135	92.113 (**p < .001**)	74.7%

Summary

Model diagnostics demonstrated that the adjusted logistic regression models had acceptable to strong performance. The CoP-based predictors (MV_EO, MV_EC, SP_EO, SP_EC) showed the highest explanatory power (Nagelkerke $R^2 > 0.52$) and excellent classification accuracy (>84%), with good calibration across Hosmer–Lemeshow tests ($p > 0.05$). Grip strength also performed strongly ($R^2 = 0.436$), followed by TUG with moderate explanatory power ($R^2 = 0.250$). FES and BBS models showed lower explanatory value ($R^2 = 0.08–0.11$) but remained relevant contributors. Importantly, no multicollinearity concerns were detected, confirming that predictors contributed independently to the models.

MV_EO, MV_EC, SP_EO, and SP_EC consistently demonstrated the strongest model performance, with pseudo R^2 values exceeding 0.50 and classification accuracy >80% in both adjusted and unadjusted models.

Grip Strength was also a reliable predictor, maintaining strong explanatory power across all models.

TUG and BBS showed moderate model strength, while FES demonstrated the weakest performance, particularly in the unadjusted model where the Hosmer–Lemeshow test was significant, indicating poor model calibration.

All other models passed the Hosmer–Lemeshow test ($p > .05$), suggesting good fit between predicted and observed outcomes.

These findings emphasize the importance of objective balance (CoP) and muscular strength measures in predicting frailty and validate the use of multivariate adjustment for improved model accuracy.
